# Effects of corticosteroid plus long-acting beta_2_-agonist on the expression of PD-L1 in double-stranded RNA-induced lung inflammation in mice

**DOI:** 10.1186/s12950-017-0149-4

**Published:** 2017-01-17

**Authors:** Saaka Hamano, Koichiro Matsumoto, Ken Tonai, Satoru Fukuyama, Keiko Kan-o, Nanae Seki, Hiromasa Inoue, Yoichi Nakanishi

**Affiliations:** 1 Research Institute for Diseases of the Chest, Graduate School of Medical Sciences, Kyushu University, Higashi-ku, Fukuoka, 812-8582 Japan; 2 Department of Pulmonary Medicine, Graduate School of Medical and Dental Sciences, Kagoshima University, Kagoshima, Japan

**Keywords:** Asthma, Viral infection, Lung inflammation, Corticosteroid, Long-acting beta_2_-agonist

## Abstract

**Background:**

Airway viral infections cause the exacerbations of asthma and chronic obstructive pulmonary disease. PD-L1, also known as B7-H1, is an immune-checkpoint molecule that plays a role in an escape mechanism of viruses from the host immune systems. This escape may be associated with the persistence of viral infection and the exacerbation of the underlying diseases. In a study in vitro, we have shown that corticosteroids plus long-acting beta_2_-agonists (LABAs) attenuate the upregulation of PD-L1 on airway epithelial cells stimulated with an analog of viral double-stranded RNA, polyinosinic-polycytidylic acid (poly I:C). To address its biological relevance in vivo, we investigated the effect of corticosteroid plus LABA on the expression of PD-L1 in double-stranded RNA-induced lung inflammation in mice.

**Methods:**

Mice were intratracheally administered with poly I:C. The expression of PD-L1 on the lung cells was assessed by flow cytometry and inflammation was assessed for bronchoalveolar lavage fluid (BALF). Independent as well as combination effects of ciclesonide and indacaterol were examined.

**Results:**

Administration of low dose poly I:C upregulated the expression of PD-L1, induced neutrophilia and increased keratinocyte-derived chemokine (KC), macrophage inflammatory protein-1β (MIP-1β), and IL-6 in BALF. The upregulation of PD-L1, neutrophilic inflammation and increase of KC were suppressed by ciclesonide plus indacaterol, but not by either when administered independently. Although the upregulation of PD-L1 by high dose poly I:C was suppressed by ciclesonide plus indacaterol, neutrophilia and increased KC, MIP-1β, and IL-6 in BALF were not attenuated.

**Conclusions:**

Ciclesonide plus indacaterol attenuate double-stranded RNA-induced upregulation of PD-L1 in the lungs.

## Background

Viral airway infections, such as rhinovirus, respiratory syncytial virus (RSV), human metapeumovirus, and influenza virus, frequently cause the exacerbation of inflammatory airway diseases including asthma and chronic obstructive pulmonary disease (COPD) [1–3]. These viruses possess single-stranded (ss) RNA as their own genome. Influenza virus has a unique tri-phosphate-ended double-stranded (ds) RNA-like structure at the 5’terminal of genomic ssRNA. Rhinovirus, RSV, and human metapeumovirus generate dsRNA in their host cells. These dsRNAs activate innate immune responses through their binding to Toll-like receptor 3 (TLR3) and the family of RNA helicase, namely, the retinoic acid-inducible gene I (RIG-I) and the melanoma differentiation-associated gene 5 (Mda5) [4–6]. TLR3 recognizes viral dsRNA and a synthetic analog of dsRNA, polyinocinic polycytidilic acid (poly I:C), in the endosome, while RIG-I and Mda5 recognize dsRNA in the cytoplasm. RIG-I recognizes dsRNA of RSV, human metapeumovirus and influenza virus. Mda5 recognizes dsRNA of rhinovirus and poly I:C. Subsequent signaling leads to adapted antiviral immunity.

Adapted immune responses are initiated by the interactions between antigen-presenting cells and T cells. These interactions are characterized by the binding of a variety of costimulatory and coinhibitory molecules, including B7-family molecules, and their putative receptors [7]. PD-L1, also named as B7-H1, belongs to the B7 family and shares its receptor, programmed death-1 (PD-1), with PD-L2, also named as B7-DC. PD-1 is induced in T cells and B cells during their activation, and its ligation to PD-L1 results in the inhibition of the activated status. PD-L1, PD-L2 and PD-1 have been known as immune-checkpoint molecules in tumor and infection immunity [8]. Previous studies showed that the blockade of the PD-L1/PD-1 pathway restored the function of virus-specific cytotoxic T lymphocytes (CTLs) and decreased the viral load, suggesting that PD-L1 upregulation causes CTL exhaustion and then impairs virus eradication [9–11]. The airway epithelial cells are targeted by viruses for their replication. Human epithelial cells constitutively expressed a low level of PD-L1 and poly I:C stimulation upregulated the expression of PD-L1 [12–14]. Given a high expression of PD-1 on virus-specific CTLs, dsRNA-induced upregulation of PD-L1 may prevent the infected cells from attack by CTLs, thereby facilitating the spread of a virus around the neighboring cells [15]. Antigen-presenting cells, particularly dendritic cells, are other important cells in the anti-viral immune defense. Virus- or dsRNA-stimulated dendritic cells trigger an array of anti-viral innate- and adapted-immune responses [16]. On the other hand, virus and dsRNA upregulates the expression of PD-L1 on dendritic cells, which may compromise the anti-viral immune responses [17, 18].

Inhaled corticosteroids (ICSs) and long-acting beta_2_-agonists (LABAs) are used worldwide for the treatment of asthma and COPD. Clinical trials showed that the combination of ICSs with LABAs improves symptoms and lung function and reduces exacerbations in subjects with asthma and COPD [19–21]. The mechanism of action for reducing exacerbation has not fully been elucidated. We previously showed that ICSs plus LABAs attenuate the poly IC- or RSV-induced upregulation of PD-L1 on airway epithelial cells in vitro [22]. To address its biological relevance in vivo, we investigated the effect of corticosteroid plus long-acting beta_2_-agonist on the expression of PD-L1/B7-H1 in dsRNA-induced lung inflammation in mice.

## Methods

### Reagents

Ciclesonide (CIC) was purchased from Santa Cruz Biotechnology (Santa Cruz, CA, USA). Indacaterol maleate (IND) was provided from Novartis Pharma AG (Basel, Switzerland). Poly I:C and dimethyl sulfoxide (DMSO) were purchased from SIGMA-ALDRICH (St. Louis, MO, USA). Anti-mouse CD16/CD32 mAb was purchased from BD Biosciences (San Diego, CA, USA). FITC-conjugated mouse anti-keratin 5/8 monoclonal Ab (mAb) was purchased from Progen (Heidelberg, Germany). Biotinylated anti-human PD-L1 mAb and anti-mouse PD-L1 mAb were purchased from eBioscience (San Diego, CA, USA). Streptavidin-phycoerythrin (SAv-PE) conjugate was purchased from BD Biosciences (San Jose, CA, USA).

### Culture of airway epithelial cells

The BEAS-2B cell line, derived from human bronchial epithelium transformed by an adenovirus 12-SV-40 virus, was cultured in 6-well plates by using DMEM/F12 containing 10% FBS, penicillin (100 U/ml), and streptomycin (100 ng/ml) at 37 °C with 5% CO_2_ in humidified air.

### Stimulation of cells with dsRNA

When cells reached semi-confluence, they were stimulated by administration of 3 μg/ml poly I:C in the culture medium. After 4 h incubation, they were replaced with a fresh medium. The cells were pretreated with CIC, IND, CIC plus IND or vehicle alone for 2 h before the stimulation. The cells were harvested 24 h after the stimulation.

### Mice

Male C57BL/6 mice (5–7 weeks old) were purchased from SLC Japan and were housed under specific pathogen-free conditions until the experiments commenced.

### Protocols

Mice were anesthetized with 80 μg/kg dexmetdetomidine i.p., subsequently with inhalation of 3–4% isoflurane. A tracheal cannulation was temporally performed with 20 gauge catheter. To elucidate time courses of PD-L1 expression and airway inflammation induced by poly I:C stimulation, mice were anesthetized and received intratracheal instillation (i.t.) of poly I:C (30 μg, in total volume of 50 μl). At 24, 72, and 168 h after poly I:C stimulation, mice were sacrificed, bronchoalveolar lavage was performed, and then the lungs were collected. Mice received i.t. of poly I:C (3 or 30 μg, in total volume of 50 μl), 4 h after the administration of CIC (3 μg, in total volume of 50 μl) and/or IND (3 μg, in total volume of 50 μl) i.t.. Poly I:C was dissolved in physiological saline before use. CIC and IND were dissolved in DMSO and diluted saline to 6% in final formulation. CIC and IND were used immediately after preparation. Sham-treated mice were used as controls. We provided ethics approval information with protocol ID (A23-048-0 and A25-007-0).

### Bronchoalveolar lavage and ELISA

After 24 h of poly I:C administration, mice were sacrificed under a mixture of ketamine and sodium pentobarbital i.p. injection, and their tracheas were cannulated via tracheostomy. The lungs were lavaged with 1 ml of PBS via tracheal cannula. Differential cell counts were performed. Samples were centrifuged at 2000 rpm for 10 min, the supernatants were collected and the concentration of keratinocyte-derived chemokine (KC), macrophage inflammatory protein-1β (MIP-1β) and IL-6 in bronchoalveolar lavage fluid (BALF) were measured using ELISA kit (BioSource, or R&D Systems).

### Flow cytometric analysis

For BEAS-2B cell study, 5 × 10^6^ cells were incubated in 100 μl of phosphate-buffered saline (PBS) with 0.5% BSA and 0.02% NaN_3_ containing biotynilated anti-human PD-L1 mAb at room temperature for 30 min. The samples were thoroughly washed and suspended in SAv-PE conjugate for 20 min. After washing with PBS/0.5% BSA/0.02% NaN_3_, the cells were fixed with 4% paraformaldehyde for 20 min. For in vivo study, lungs were cut and minced. PBS containing 2 mM dithiothreitol, 5 mM EDTA was added to the minced lungs and incubated for 40 min at 37 °C. The samples were filtered through a 70 μm pores size nylon cell strainer. The single-cell suspensions were washed with complete PBS, and the erythrocytes were lysed with an NH_4_Cl-Tris buffer. Cells were suspended in PBS with 0.5% BSA and 0.02% NaN_3_, and 0.02%EDTA, referred as a washing buffer. Cells were preincubated with anti-mouse CD16/CD32 mAb to prevent nonspecific binding via FcγR. For the dual-color flow cytometry, cells were incubated with FITC-conjugated anti-keratin 5/8 mAb for 30 min, followed by the addition of biotinylated anti-mouse PD-L1 mAb for 30 min. The samples were washed with the washing buffer and suspended in SAv-PE conjugate for 20 min. Cells were thoroughly washed, fixed with 4% paraformaldehyde, and flow cytometric analysis was performed using a FACSCalibur with CellQuest software (BD Biosciences, CA, USA). Thirty thousand events were acquired in a list mode with debris excluded by the forward-scatter threshold. The mean fluorescence intensity (MFI) was compared with control staining using an irrelevant isotype-matched mAb.

### Data analysis

Values were expressed as the means ± SEM. Differences among groups were analyzed using an ANOVA followed by the multiple comparison test of Tukey-Kramer. JMP Pro 11 software was used for statistical analysis. P-values less than 0.05 were accepted as statistically significant.

## Results

**Fig. 1:**
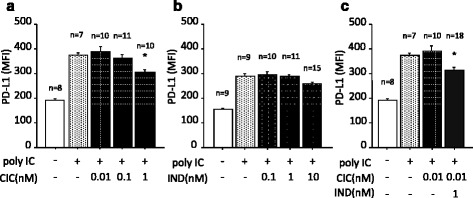
Poly I:C-induced upregulations of PD-L1 on BEAS-2B cells were suppressed by low-concentration of CIC plus IND, but not by either when administered independently. Cells were pretreated with CIC (**a**), IND (**b**), and CIC + IND (**c**) for 2 h and administered with poly I:C (3 μg/ml) to the culture medium for 24 h, and then processed for flow cytometric analysis of PD-L1. *p < 0.05 compared with stimulated control. Data is expressed as the means ± SEM of 7–10 experiments

The administration of poly I:C to the cell culture medium significantly upregulated the expression of PD-L1 on BEAS-2B cells. The upregulation of PD-L1 was suppressed by CIC alone in a concentration-dependent manner, and the upregulation of PD-L1 was significantly suppressed by CIC at 1 nM (Fig. 1a). On the other hand, IND alone had no effect on the upregulation of PD-L1 (Fig. 1b). Although CIC at 0.01 nM had no effect, CIC at 0.01 nM in combination with IND at 1 nM significantly suppressed the upregulation of PD-L1 (Fig. 1c).

**Fig. 2:**
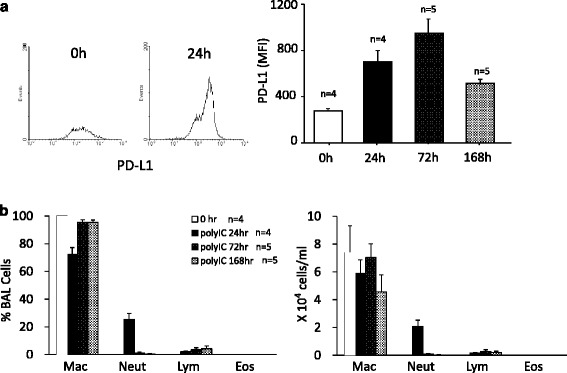
Intratracheal instillation of poly I:C induced upregulation of PD-L1 in the lung cells and neutrophilic inflammation in mice. Mice were anesthetized and received intratracheal instillation (i.t.) of poly I:C, 30 μg. At each time point, mice were sacrificed, bronchoalveolar lavage was performed, and then the lungs were collected. The lung cell suspensions were stained with FITC-conjugated anti-keratin 5/8 mAb and biotinylated anti-mouse PD-L1 mAb, followed by SAv-PE conjugate, and then processed for flow cytometry. The representative histograms of PD-L1 on keratin 5/8 mAb-positive cells in the lungs are shown and assessed by the mean florescence intensity (MFI). **a** Differential cell counts for bronchoalveolar lavage fluid were performed (**b**). *p < 0.05 compared with stimulated control. Data is expressed as the means ± SEM of 4–5 mice

For in vivo experiments, administrations of poly I:C, 3 and 30 μg, were used as preclinical models of light and heavy infections, respectively. It is known that anti-keratin 5/8 mAb has been used for detecting epithelial cell lineages in various organs including airway epithelium [23–25]. In a preliminary study, we found that this mAb also reacts with a part of CD45-positive myeloid-derived cell lineages. Single saline (i.t.) administration had no effect on the expression of PD-L1 in keratin 5/8 mAb-positive cells in flow cytometry. The administration of poly I:C, 30 μg, significantly upregulated the expression of PD-L1 compared to the sham-treated control (Fig. 2a). The upregulation of PD-L1 was apparent at 24 to 72 h after poly I:C administration. Poly I:C-induced inflammation was assessed by differential cell counts in BALF. The administration of poly I:C, 30 μg, induced neutrophilia in BALF compared to the sham-treated control (Fig. 2b). The neutrophilia was shown 24 h after poly I:C administration but rapidly disappear thereafter. In order to evaluate drug effects on the expression of PD-L1 and neutrophilic inflammation simultaneously, the time point at 24 h after poly I: C was chosen for subsequent experiments.

**Fig. 3:**
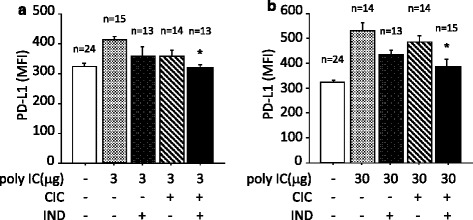
Poly I:C-induced upregulations of PD-L1 on lung cells were suppressed by CIC plus IND, but not by either when administered independently. Mice were anesthetized and received intratracheal instillation (i.t.) of poly I:C, 3 μg (**a**) or 30 μg (**b**). Mice were sacrificed and the lungs were collected. The lung cell suspensions were stained with FITC-conjugated anti-keratin 5/8 mAb and biotinylated anti-mouse PD-L1 mAb, followed by SAv-PE conjugate, and then processed for flow cytometry. CIC (3 μg) and/or IND (3 μg) i.t. were administered 4 h before poly I:C stimulation. The drug effects were evaluated 24 h after poly I:C stimulation, *p < 0.05 compared with stimulated control. Data is expressed as the means ± SEM of 13–24 mice

Fluticasone/salmeterol or budesonide/formoterol was used for our previous study in vitro [22]. In the present study in vivo, CIC/IND was chosen because their drug half-lives are longer than those used for the previous study, which was adequate for our proposal that drug efficacy should persist over a day in this protocol. Although the poly I:C (3 μg i.t.)-induced upregulation of PD-L1 was not affected by CIC or IND when used independently, it was completely suppressed by treatment with CIC plus IND (Fig. 3a). The administration of poly I:C, 30 μg, markedly upregulated the expression of PD-L1, which was not affected by CIC or IND alone. The upregulation was significantly suppressed by CIC plus IND (Fig. 3b).

**Fig. 4:**
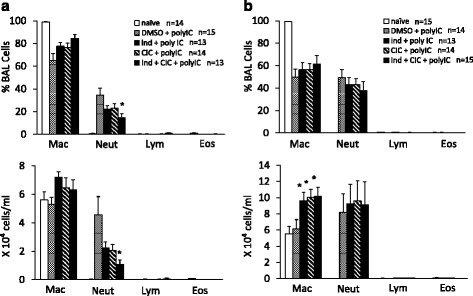
Low-dose poly I:C-induced neutrophilic inflammation were suppressed by CIC plus IND, but not by either when administered independently. Mice were anesthetized and received poly I:C, 3 μg (**a**) or 30 μg (**b**) i.t., 4 h after the administration of CIC (3 μg) and/or IND (3 μg) i.t.. 24 h after poly I:C administration, bronchoalveolar lavage fluid was collected and differential cell counts were performed. *p < 0.05 compared with stimulated control. Data is expressed as the means ± SEM of 10–20 mice

Although the poly I:C (3 μg i.t.)-induced neutrophilia was not affected by CIC or IND alone, it was significantly suppressed by treatment with CIC plus IND (Fig. 4a). The administration of poly I:C, 30 μg, induced marked neutrophilia. The neutrophilia was not affected by CIC or IND alone or CIC plus IND (Fig. 4b). Poly I:C, 30 μg, did not affect the number of alveolar macrophages in BALF but the number was significantly increased by CIC, IND, and CIC plus IND, when used both independently and as a combination. There was no significant difference among those three groups.

**Fig. 5:**
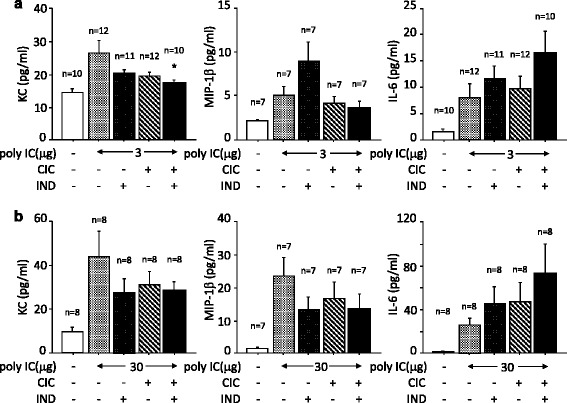
Low-dose poly I:C-induced increases in KC in BALF were suppressed by CIC plus IND, but not by either when administered independently. Effects of CIC and IND used independently and their combination on airway inflammation induced by poly I:C stimulation of mice. Mice were anesthetized and received poly I:C, 3 μg (**a**) or 30 μg (**b**) i.t., 4 h after the administration of CIC (3 μg) and/or IND (3 μg) i.t.. Twenty four hours after poly I:C administration, bronchoalveolar lavage fluid was collected and ELISA of KC, MIP-1β and IL-6 were performed. *p < 0.05 compared with stimulated control. Data is expressed as the means ± SEM of 6–12 mice

The administration of poly I:C, 3 μg, increased the concentration of KC in BALF compared to the sham-treated control. Although the increase was not affected by CIC or IND when used independently, it was significantly suppressed by treatment with CIC plus IND (Fig. 5a). The administration of poly I:C, 30 μg, also increased the concentration of KC. The increase was not significantly affected by independent uses of CIC, IND, or CIC plus IND (Fig. 5b). The administration of poly I:C, 3 and 30 μg, increased the concentrations of MIP-1β and IL-6 in BALF compared to the sham-treated controls. The increases were not significantly affected by either CIC or IND or CIC plus IND.

## Discussion

In the present study, combination effects of CIC/IND were examined for the expression of PD-L1 and inflammation in dsRNA-induced models of airway viral infection. We postulated that two doses of poly I:C could reflect light and heavy infection mainly based on RSV-infection in humans. It is well known that a proportion of infants with RSV infection develop severe bronchiolitis whereas a majority of RSV-infected infants and healthy adults have limited disease such as cold-like symptoms. Although the precise mechanisms explaining those different patterns of outcomes remain uncertain, several studies suggested that neutrophilic inflammation may be readout of viral loads [26, 27]. A part of them may be derived from net burden of viral dsRNA. There have been several studies that used poly I:C in order to mimic airway viral infection [28–30]. These studies showed that a single topical administration of poly I:C to the respiratory tracts dose-dependently induced neutrophilic airway inflammation and increased KC, MIP-1β, and IL-6 in BALF, which was also confirmed in the present study. In the experiments of poly I:C (3 μg i.t.) as a model of light infection, the upregulation of PD-L1, neutrophilic inflammation and the elevation of KC were significantly suppressed by CIC plus IND, but not by CIC or IND alone. Given that KC is a strong chemoattractant for neutrophils, the reduction of neutrophilic inflammation may be the outcome of reduced production of KC by CIC plus IND. In the experiments of poly I:C (30 μg) as a model of heavy infection, the upregulation of PD-L1 was also suppressed by CIC plus IND but the neutrophilic inflammation and the productions of KC, MIP-1β, and IL-6 in BALF were not attenuated. These results suggest that the suppression of PD-L1 by CIC plus IND was not due to reduced impact of poly I:C-induced neutrophilic inflammation. We did not measure PD-1 because methodological limitation in this study. The use of poly I:C, a synthetic double-stranded RNA, may mimic virus-induced innate immune responses but not induce viral antigen-induced adoptive immune responses including viral antigen-specific CTLs expressing PD-1. We also did not measure PD-L2 because pathophysiological roles of PD-L2 in airway viral infection remain uncertain.

The synergy of CIC/IND may be partly explained by its direct effect on epithelial cells according to the results obtained from the study in vitro using cultured BEAS-2B cells. In the study in vivo, the expression of PD-L1 was assessed for keratin 5/8 mAb-positive cells that include epithelial cells and myeloid-derived inflammatory cells. The myeloid-derived dendritic cells increase their expression of PD-L1 in response to dsRNA [17, 18, 31]. Recent investigations revealed that there are different neutrophil subsets in mice and humans [32–34]. A CD16^high^/CD62L^dim^ subset is known to suppress lymphocyte proliferation and induces lymphocyte apoptosis, through PD-L1 expression on this subset [33, 34]. These studies raise a possibility that suppression of PD-L1 expression in CIC/IND-treated mice may be partly due to downregulation of PD-L1 on the neutrophil subset.

Although there has been no literature showing the effect of ICS plus LABA on virus-induced upregulation of PD-L1 in vivo, a previous study showed that treatment of RSV-infected mice with fluticasone propionate plus salmeterol resulted in a 50% decrease of RSV titer in the lung and a reduction in neutrophils compared to either of drugs alone [35]. The decrease of viral titer by ICS plus LABA may affect the expression status of PD-L1 in real viral infection. The presented model has a technical limitation. Viruses can amplify themselves within several days of initial airway infection, whereas poly I:C can be eliminated by the lung soon after administration, making it difficult to elucidate sub-chronic immune responses to viral infection.

It is important to consider the outcome of PD-L1 suppression by ICS/LABA on airway viral infection. The upregulation of PD-L1 on epithelial cells render virus-specific CTLs to fail to eradicate virus-infected epithelial cells [14, 15]. The upregulation of PD-L1 on myeloid-derived inflammatory cells, particularly the neutrophil subset described above, suppress lymphocyte proliferation, induce lymphocyte apoptosis, and then compromise anti-pathogen host defense [34, 36]. With this regard, ICS/LABA treatment may be beneficial for earlier virus eradication. On the other hand, a recent study reported that PD-L1 upregulation on a subset of dendritic cells prevents excessive immune reaction triggered by RSV [18]. Several studies provided novel information regarding the expression kinetics of PD-L1 in microorganism infections. One study showed that influenza virus-induced expression of PD-L1 was decreased on macrophages in subjects with COPD compared with those in control subjects [37]. The other study showed that early-life Chlamydia respiratory infection-induced PD-L1 promoted severe inflammation during inflammation, permanent reductions in lung function, and the development of more severe allergic airway disease in later life in a mouse model of lung inflammation [38]. As for human rhinovirus, the most common virus linked to exacerbation of asthma and COPD, human airway epithelial cells infected with the virus upregulated the expression of PD-L1 [13]. These studies suggest the diversity in the expression kinetics of PD-L1 and then the impacts of infection-induced upregulation of PD-L1 may be different among underlying diseases. Further investigation is required to discriminate PD-L1-expressing cell types in the lungs and to evaluate ICS/LABA on PD-L1 expression on each cell type.

## Conclusions

Administration of poly I:C upregulated the expression of PD-L1, induced neutrophilia and increased KC, MIP-1β and IL-6 in BALF. The upregulation of PD-L1, neutrophilic inflammation and increase of KC were suppressed by ciclesonide plus indacaterol, but not by either when used independently of each other.

## Abbreviations

BALF: Bronchoalveolar lavage fluid
CIC: Ciclesonide
COPD: Chronic obstructive pulmonary disease
CTLs: Cytotoxic T lymphocytes
dsRNA: Double-stranded RNA
ICSs: Inhaled corticosteroids
IND: Indacaterol maleate
KC: Keratinocyte-derived chemokine
LABAs: Long-acting beta_2_-agonists
mAb: Monoclonal antibody
MFI: The mean fluorescence intensity
MIP-1β: Macrophage inflammatory protein-1β
poly I:C: Polyinosinic-polycytidylic acid
SAv-PE: Streptavidin-phycoerythrin
